# Hybrid AC/DC architecture in the CE.D.E.R.-CIEMAT microgrid: demonstration of the TIGON project

**DOI:** 10.12688/openreseurope.15154.2

**Published:** 2024-01-09

**Authors:** Paula Peña-Carro, Oscar Izquierdo-Monge

**Affiliations:** 1 Energy, Centro de Investigaciones Energéticas, Medioambientales y Tecnológicas, MADRID, SPAIN, 28040, Spain

**Keywords:** Hybrid microgrid, DC microgrid, direct current (DC), DC/AC conversion, DC/DC converter, MVDC (medium voltage DC), LVDC (low voltage DC)

## Abstract

This article presents the demonstrative development of the Towards Intelligent DC-based hybrid Grids Optimizing the Network performance (TIGON) project at the Centre for the Development of Renewable Energy - Centre for Energy, Environmental and Technological Research (CE.D.E.R.-CIEMAT), as well as the established objectives to be achieved with the implementation of a microgrid with smart grid architecture based on direct current (DC) and integrated into the current energy system. This type of architecture is proposed as a future solution to reduce energy losses caused by DC-alternating current (AC) conversions, increasing the overall performance and profitability of hybrid grids. All this without forgetting to ensure the supply, stability and reliability of the system with the development of all the necessary equipment and protections to make this approach a reality. The microgrid design and process of implementation start from a transformation centre, from which the medium voltage direct current (MVDC) grid will be created by the Solid State Transformer (SST). In the MVDC grid, we will find a bank of lead-acid batteries and other essential equipment in the microgrid, a DC/DC converter that will create the low voltage direct current (LVDC) grid. On the LVDC side, several branches have been designed to connect the rest of the systems; generation (mini-wind and photovoltaic), storage (LFP batteries) and loads (AC and DC loads). Each of the equipment will have a connection to the DC grid through converters made exclusively for this equipment and connexion to the AC grid, which will allow us to obtain all the necessary data to carry out the required studies to achieve the established objectives of the project.

## Plain language summary

Most of the current electrical grid infrastructure is based on alternating current (AC) because the facilities used for long distance power distribution are made to operate in AC. The reason behind this is that power losses are less at high voltage AC. However, in recent years, renewable energies, local generation and consumption are being promoted ^1^, which is leading to an important energy transition for all citizens.

This transition also derives from the type of current generated from renewable energy sources (such as solar or wind power, these being the most widespread technologies), as the vast majority of them generate energy in direct current (DC) ^2^. In addition, it is common to find storage systems associated with these renewable energy sources, due to their variability linked to weather conditions. These storage systems also operate in DC. Finally, to close the generation, storage and consumption cycle, part of the consumption with devices used on daily basis is carried out in DC, such as LED lighting, computers or mobile phones use DC for their operation. Other examples include charging stations for electric vehicles, which are becoming more and more widespread, and the electric railway system ^3, 4^. This implies achieving conversions in current types resulting in losses linked to these changes ^5– 8^ and thus reducing the amount of energy reaching the consumer versus the amount of energy produced.

TIGON project seeks to facilitate this transition and bring it to reality by generating equipment, allowing the transition to DC, and by reducing AC consumption. It therefore benefits us as consumers, thanks to the reduction of energy conversion losses associated with the transformation from AC to DC. CE.D.E.R.-CIEMAT, as a demonstration centre for the project, will have a DC-based hybrid microgrid where this idea can be integrated and operated in a real location.

## Introduction

Microgrids are characterised as a network with clearly defined limits managed as a single system, in which we find different sources of distributed generation, storage and consumption systems ^9^. One of the benefits associated with this way of operating is the use of local resources, managing to reduce energy transport distances and thus the losses related to them, improving the generation-consumption energy efficiency.

These distributed generation sources are mostly of renewable origin, the best known and most widely used being wind and photovoltaic systems. These types of technologies generate direct current (DC) ^10– 12^, either directly or through a power converter. Another element that operates in DC, highlighting its importance within the microgrid due to the intermittency associated with renewable energy sources, is the storage systems, which play a significant role in balancing load and power within the DC microgrid ^13, 14^. Thus, we observe that the predominant operation within a microgrid is in DC, versus alternating current (AC) operation.

Modern electrical equipment, including computers, mobile phones, ventilation systems, electric vehicles, etc. ^15^, are also used in the DC mode of operation. In contrast, we find that most of the infrastructure of the electricity grid is centralised and is operated in AC. The reason for its use in this type of current is the reduction of electricity losses at high voltages during transport compared to DC. Due to this disparity in the type of current between the generation mode and consumption mode, it is necessary to convert from DC to AC by using a DC/AC converter in order to be able to use the equipment that is plugged into the mains. This conversion produces energy losses ^16^, both in the converter, producing a total energy loss of approximately 10–25% ^13^, and in the transport from the point of generation to the point of consumption. In turn, they reduce the efficiency of electrical systems.

These disadvantages associated with AC consumption make DC grids attractive in the energy sector ^17^. This is largely due to the increase in DC loads, such as in LED lighting, electric vehicle charging stations, energy storage, etc. It is also due to the increasing growth of distributed generation sources, where the transport of electricity is significantly reduced ^18^.

The main factors driving this electricity paradigm shift are related to the improved efficiency, flexibility, safety and reliability that hybrid AC/DC grids can provide, thus increasing the sustainability of the power distribution system ^11, 19, 20^.

The TIGON (Towards Intelligent DC-based hybrid Grids Optimizing the Network performance) project is framed within the European Union, financed by Horizon 2020. In total, 15 entities from eight European Union countries are participating. Among them is the Centre for the Development of Renewable Energy (CE.D.E.R.), which is one of the demonstrators of the project.

TIGON was created to demonstrate the possibilities offered by microgrids with hybrid architectures compared to their AC counterparts, as cited in various studies referring to improved economics, reduced conversion losses, low line losses, high efficiency, resilience and reliability ^1, 11, 21^. It is also intended to consolidate all the control systems, topologies and equipment developed in the project. With the aim that these solutions can move from being a promising solution for future smart grids to a commercially available technological option.

The DC microgrid proposes a four-level approach aimed at improving reliability, resilience, performance and cost-efficiency through the development of power electronics solutions, systems and software tools focused on the efficient monitoring, control and management of DC grids.

The study is organised in sections as follows: Firstly, it presents the CE.D.E.R. centre where the hybrid architecture microgrid is located. Next, it explains the details of the monitoring system implemented in the centre for the follow-up of the different equipment. Finally, it describes the future work planned with this project.

## Location: CE.D.E.R. - Centre for Energy, Environmental and Technological Research (CIEMAT)

One of the project's demonstrators is the CE.D.E.R. centre in Lubia (Soria, Spain). This public research organisation is part of the CIEMAT centre and is attached to the Department of Energy. The centre specialises in the development and promotion of renewable energies. It has extensive facilities for scientific and technological demonstrations. For these reasons, it is an ideal environment for the installation and study of the microgrid project with a DC grid.

The centre can be considered a microgrid, with different generation systems, such as photovoltaic panels, wind turbines, biomass, mini-hydro, storage systems (lithium ferro-phosphate [LFP] batteries and lead-acid batteries) and different loads. All of this are controlled and monitored in real-time through an interface developed by us. Through this interface we also have the capacity to switch on and off all the equipment instantaneously and to vary the power of those that allow it, such as the mini-hydraulics or the storage systems.

The general microgrid of the CE.D.E.R. centre has a medium voltage grid (15 kV) in which eight transformer substations can be found that adjust the voltage from 15 kV to 400 V three-phase low voltage. For the TIGON demonstration, we will focus on an area within the centre's facilities called PEPA II. It will host the microgrid with DC-based grid architecture connected via a solid-state transformer (SST). The SST is developed exclusively for this project by one of the consortium members. It will have the function of connecting the main grid of the centre with the DC microgrid. Through this point of common coupling (PCC), which connects LVAC with MVDC, the microgrid will be able to operate in isolation or connected to the distribution network.

We are currently in the second year of the project. Different equipment is being developed simultaneously, such as the solid-state transformer, the silicon carbide (SiC) medium voltage DC/DC power converters, and the energy management system, wide area monitoring protection and control (WAMPAC), together with the development of a cybersecurity system.

At the same time, the project demonstrators, in this case the CE.D.E.R. centre, are adapting their facilities to host all the equipment.

**Figure 1.**
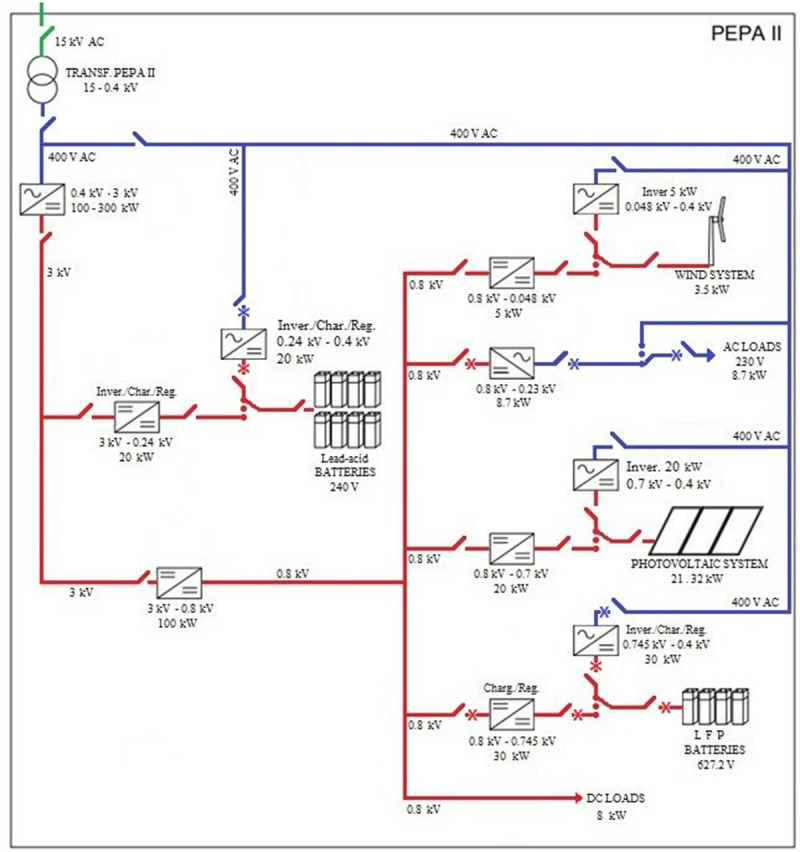
Diagram of alternating current (AC)/direct current (DC) hybrid microgrid architecture.

Figure 1 shows the organisation and composition of all the elements that configure the hybrid AC/DC microgrid in PEPA II.

Apart from the devices under development, the centre has generation and storage systems that are currently installed and in operation.

We are collecting data on their operation for subsequent analysis. This will serve as a comparison between production and consumption in an AC network and, later, in a DC network.

Thanks to these records with the same equipment but in different operating scenarios, referring to DC/AC, we will be able to carry out different studies. These will be studies of production yields, costs, benefits, energy losses due to conversions and equipment performance, thus eliminating factors such as, for example, the difference in equipment or different locations with different productions influenced by local resources.

Following are the details of all the installed components that make up the project.

The green line refers to the 15 kV alternating current that reaches the centre's installations and to the voltage fed to the transformer substation, which is responsible for generating the 400 V alternating current that feeds the hybrid microgrid.

If we look at the DC part (red line), we find a medium voltage grid containing the Lead-acid batteries; and a low voltage grid housing the photovoltaic system, wind turbine, LFP batteries, and loads.

On the AC side (blue line), there are the AC loads of the centre, such as offices, laboratories, computers, etc.

### Generation elements

Two technologies have been installed in the low-voltage part of the project: a wind and a photovoltaic system.

**Figure 2.**
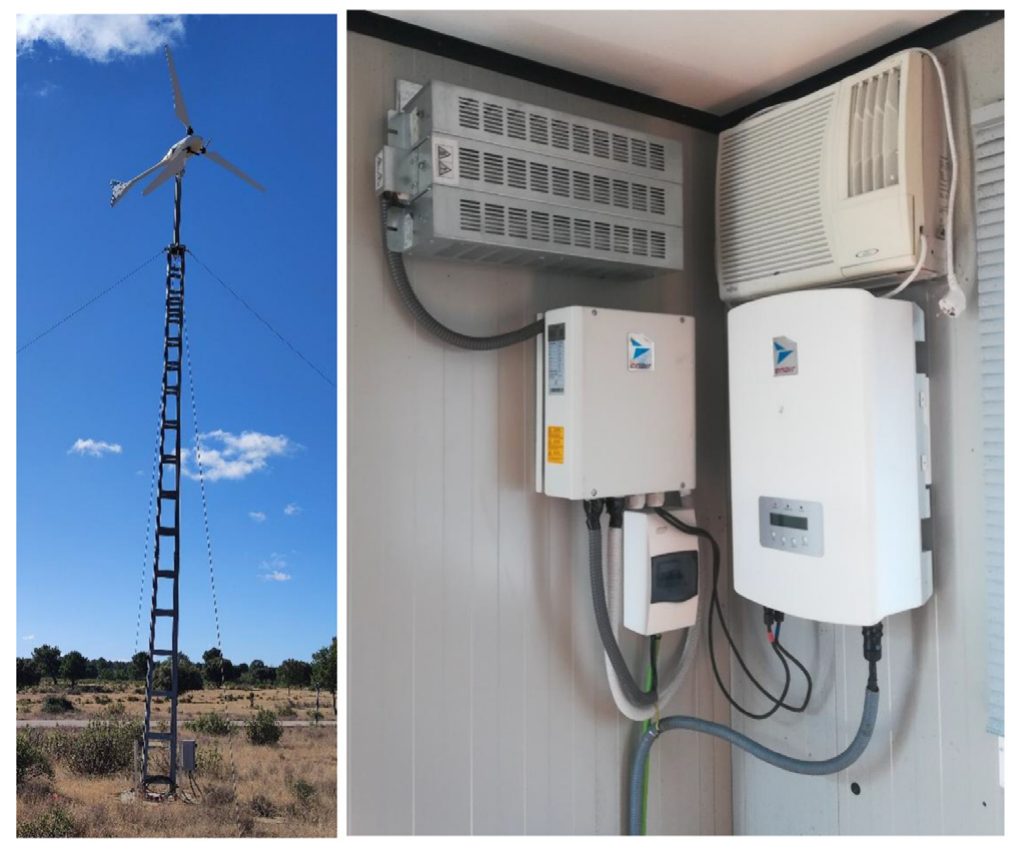
Small wind system.

Figure 2 shows the small wind system. It consists of a Ryse Energy E5 three-bladed small wind turbine, with a rated output of 3.5 kW windward horizontal axis. For AC operation, the AC inverter must be incorporated into the system, together with a dissipative resistor and the brake, which are part of the wind turbine's safety system.

**Figure 3.**
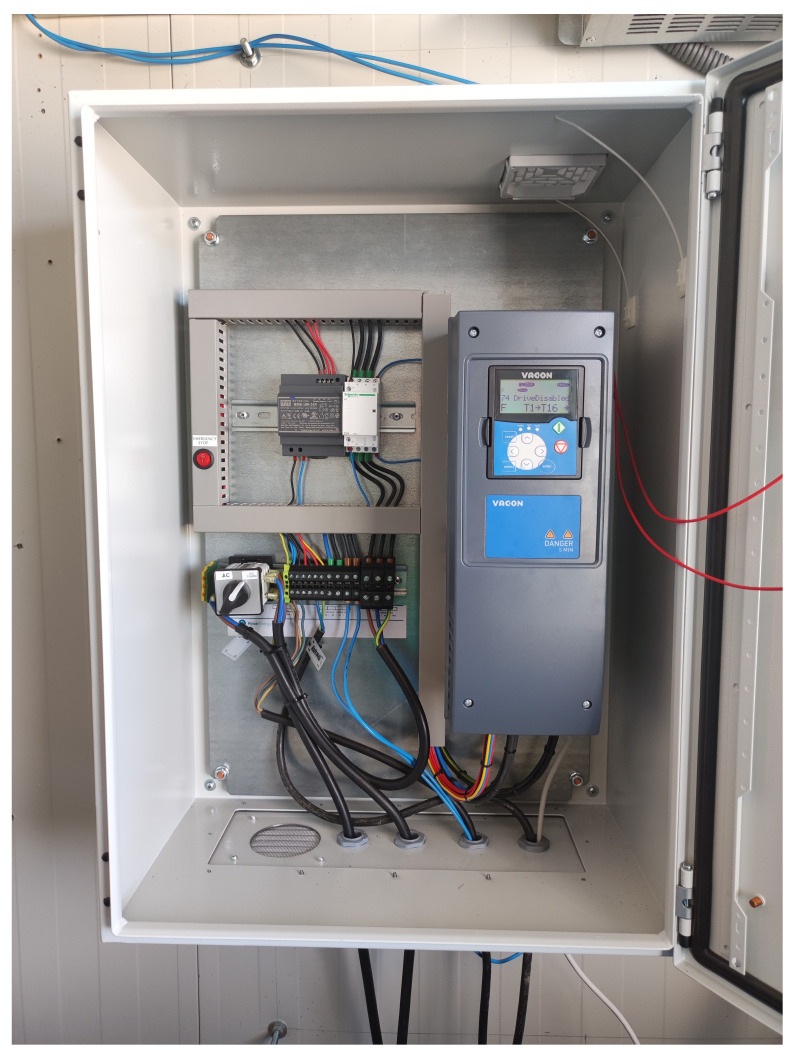
Direct current/direct current (DC/DC) wind converter.

With all the equipment mentioned in the previous section, we would have AC operation covered, but we need a DC-DC converter for DC operation (see Figure 3). This equipment adapts the voltage of the wind turbine (0.048 kV) to the DC grid voltage (0.8 kVdc).

Inside the box there is a selector switch that allows you to manually select the desired operating mode, either AC or DC.

**Figure 4.**
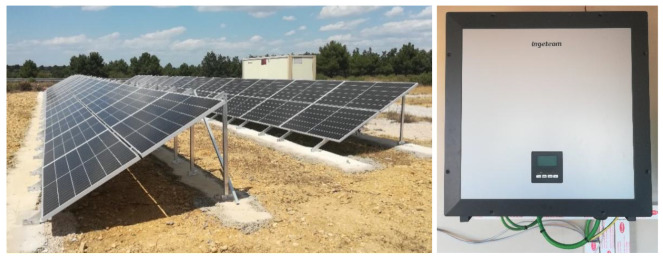
Photovoltaic system.

Figure 4 presents the photovoltaic system, consisting of 52 monocrystalline silicon panels of 410 W in a series-parallel arrangement. Connected to an Ingecon Sun 3 Play inverter with a nominal power of 20 kW, which allows us to operate in AC.

**Figure 5.**
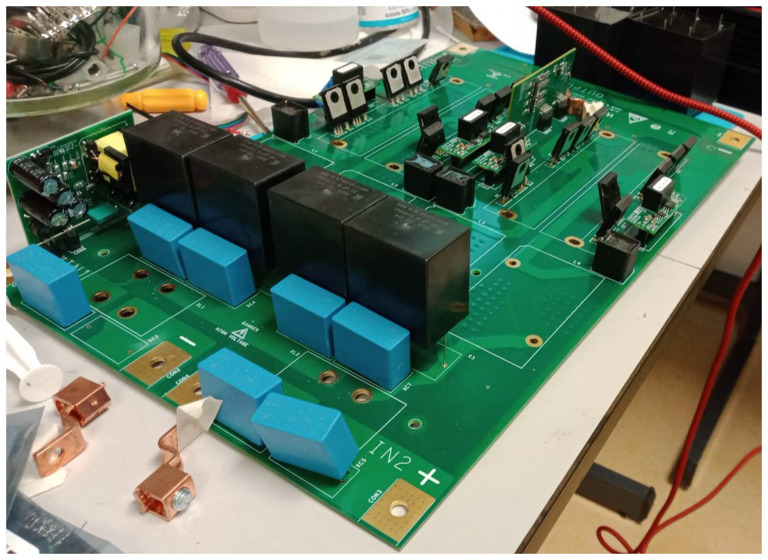
Direct current/direct current (DC/DC) converter for photovoltaic (PV) system in development.

To complete the system and include it in the proposed hybrid microgrid, the University of Valladolid (UVa), together with our centre, is developing a DC/DC converter that will allow operation at 0.8 kVdc thanks to the voltage and current adaptation carried out by the equipment (see Figure 5).

### Storage systems

The centre has two electrochemical storage systems. The first one is a bank of Lead-acid batteries located on the MVDC. The other system is a bank of LFP batteries on the LVDC.

**Figure 6.**
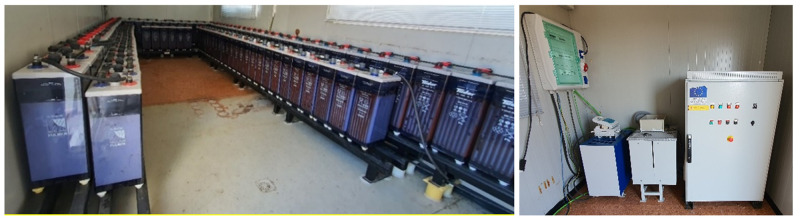
Lead-acid battery system.

Figure 6 shows the Lead-acid battery system: battery bank consisting of 120 cells of 2 V each, with a capacity of 1080 Ah (C _120_) and a total voltage of 240 Vdc. In the picture on the right, we can see all the equipment for the operation of the AC system. Citing them from right to left, DC/AC inverter of 30 kW, capacitor bank and inductance to compensate the reactive start-up power, and electrical panel of the system.

The DC/DC inverter, capable of adapting the MVDC line voltage (3 kVdc) to the battery voltage (240 Vdc), is being developed by two members of the project consortium. This SiC equipment will allow the installation of a system in the medium voltage branch of the designed DC grid, which in our case, will be the only system at this operating voltage.

**Figure 7.**
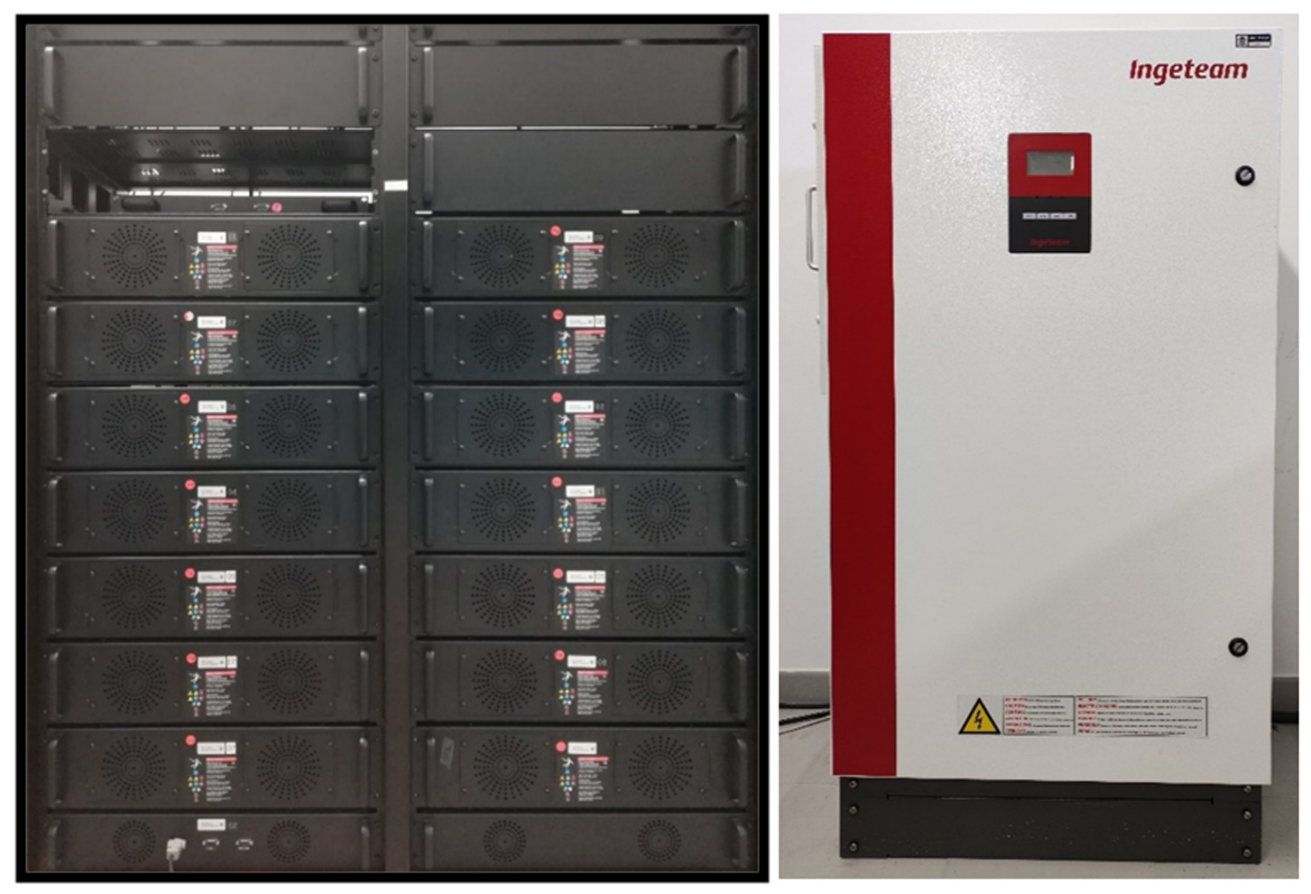
Lithium ferro-phosphate (LFP) battery system.

Figure 7 shows the LFP battery system: made up of 14 modules, with 14 cells of 3.2 V each, achieving a total voltage of 627.2 Vdc and a nominal capacity of 50 Ah. Managed via a BMS and an Ingecon Sun 30 inverter adapted for this function.

The operation of these batteries on the LVDC line will be possible with the installation of the DC/DC inverter which, with the power electronics designed by a team of researchers from UVa and our centre, manages to increase the system voltage up to 800 VDC.

**Figure 8.**
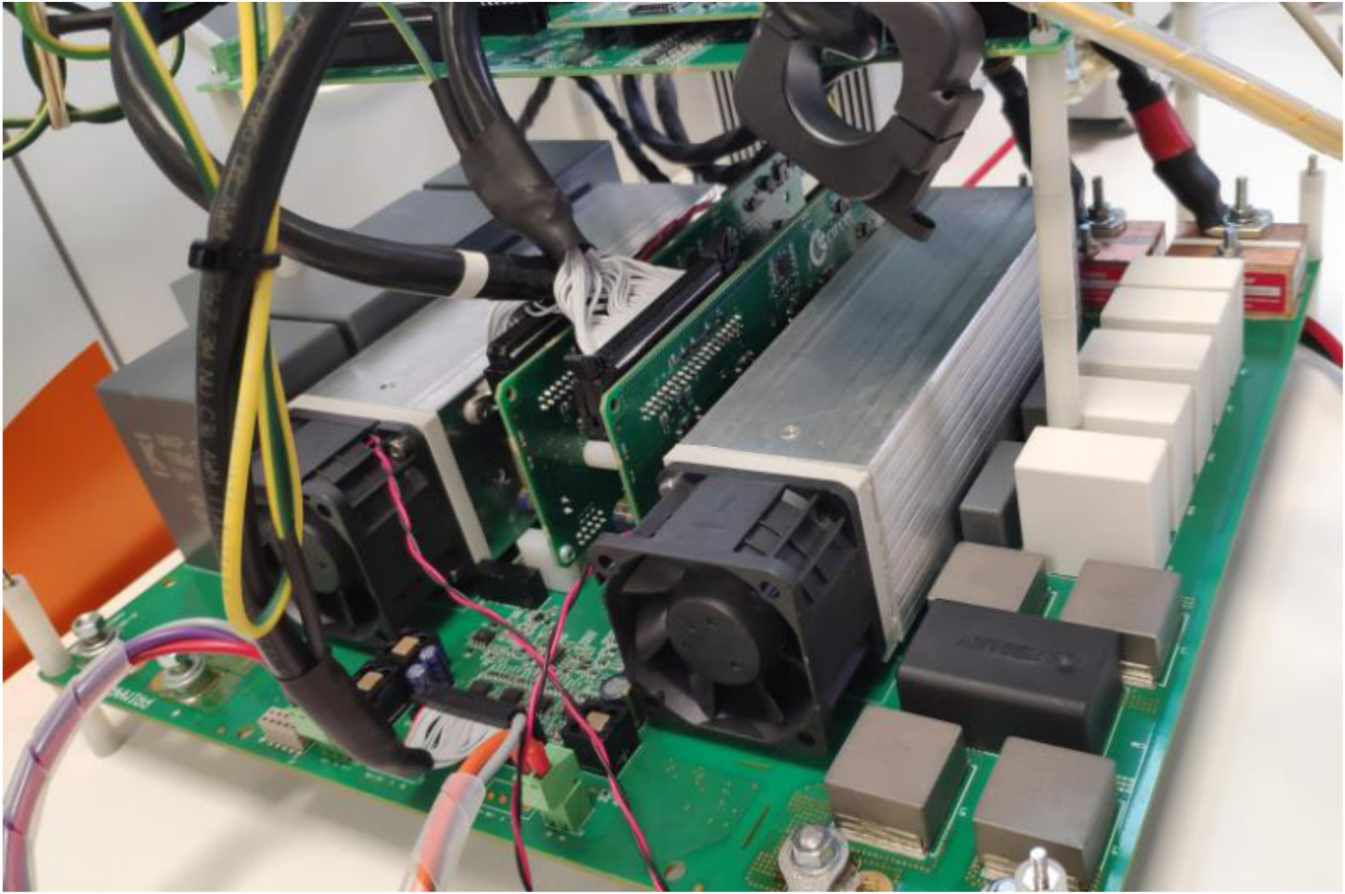
Direct current/direct current (DC/DC) lithium ferro-phosphate (LFP) converter.

This equipment is currently under development as can be seen in the Figure 8.

### Loads

We distinguish three loads. The first of these is the microgrid of the CE.D.E.R. in AC (lights, computers, machinery, laboratory processes, etc.), located upstream of the transformer substation, which is the largest load in terms of power.

The remaining two loads are programmable, one located on the AC grid and the other on the DC grid.

**Figure 9.**
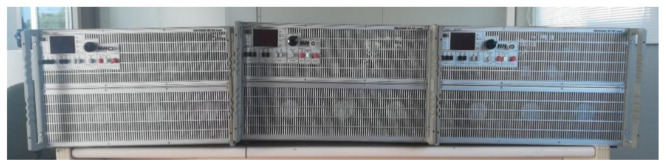
Programmable alternating current (AC) loads.

As for the AC programmable loads, Figure 9 shows three AC2928 programmable loads whose working mode is master-slave with a power of 2.9 kW each. We have one master and two slaves. Thanks to their programmability (daily and hourly), they allow us to set operating periods coinciding with different desired consumption patterns. To couple its operation to the proposed microgrid, a company that develops power electronics equipment in the DC/DC field is in charge of making this equipment.

**Figure 10.**
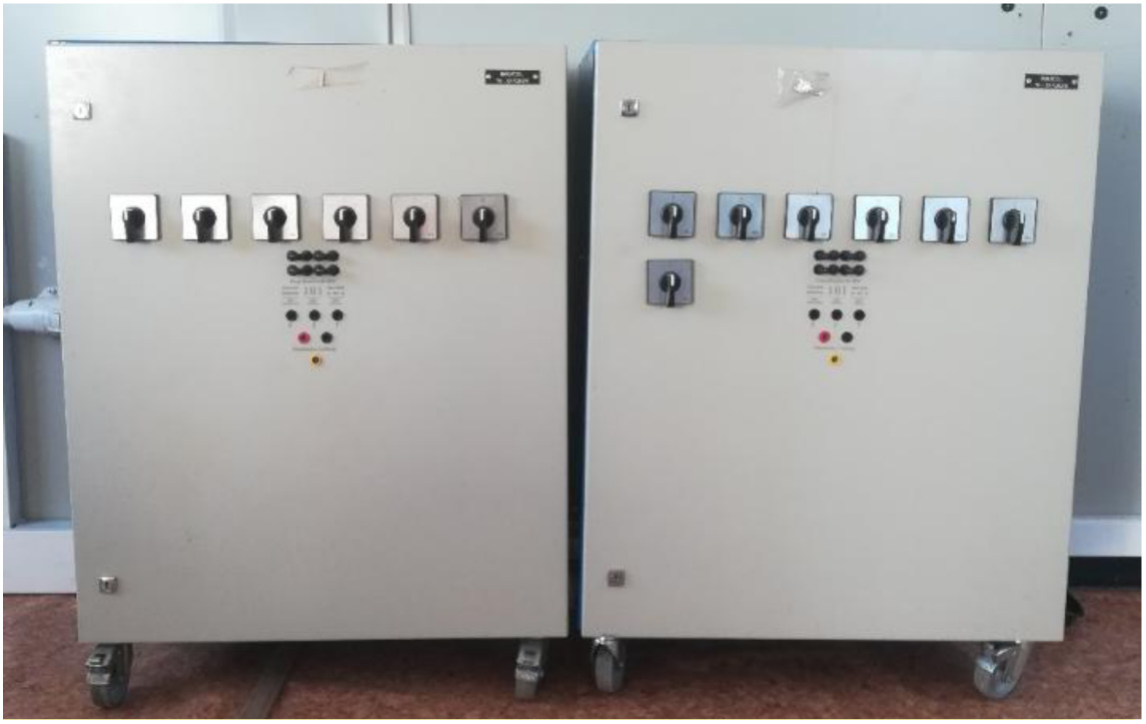
Programmable direct current (DC) loads.

Regarding the continuous loads, there are two 4 kW Enelec resistive loads installed (see Figure 10), which allow the possibility of working in both three-phase and continuous, depending on the required needs. In our case, we will opt for DC consumption, which can be manually applied to different percentages of variation of the total power of the loads, thereby achieving the application of different consumptions and seeing how the microgrid responds.

## MVDC/LVDC power converter and solid-state transformer (SST)

This section focuses on two of the most important power electronics equipment within the hybrid microgrid structure proposed for this project. These devices are more important than the rest because without them it would not be possible to create the medium voltage grid, nor the low voltage DC grid, which is our main objective.

The DC/DC converter and the SST are therefore a fundamental part of the development of the project. This task is being carried out by two members of the consortium under several premises.

The material used is SiC and its development, apart from being functional for final applications, is intended to have flexible configurations in order to provide flexibility and replicability to the equipment so that these designs are suitable for future hybrid microgrids by applying simple modifications. In this way, its development is proposed in several power electronics building blocks (PEBB) that can be joined in series/parallel to achieve the desired voltage and nominal power levels.

Solid State Transformer, equipment under development by the Centre for Research on Energy Resources and Consumption (CIRCE). This equipment will have the capacity to transform the 400 V in AC received from the transformation centre to 3 kV in DC, the point from which the entire DC-based microgrid architecture will start.

**Figure 11.**
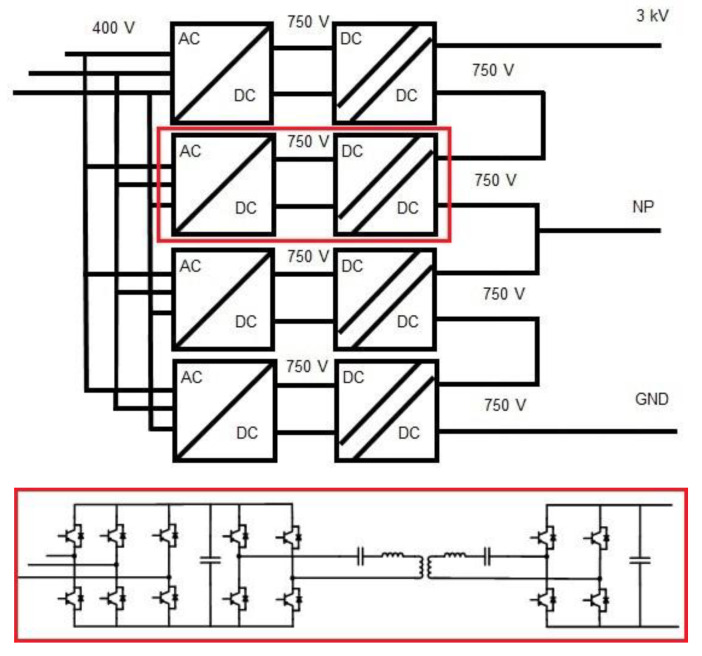
Solid-state transformer (SST) design details.

Some details of its design can be seen in Figure 11.

The other equipment mentioned in this section is the DC/DC converter, which will adapt the grid voltage from MVDC to LVDC. This device is not directly connected to any generation, storage or consumption system of the microgrid, but it will enable all the elements mentioned above to be connected.

Thanks to the design, which is currently being developed by the French Alternative Energies and Atomic Energy Commission (CEA), one of the members of the project, we will be able to adapt the 3 kV voltage to 0.8 kV with a power of 100 kW capable of taking on all the systems that will be connected downstream.

## Microgrid monitoring

Monitoring the grid let us see what happens in the microgrid in real-time and make immediate decisions to improve the performance of both individual devices and the overall microgrid. This is the main objective of the Wide Area Monitoring Protection and Control (WAMPAC) system: to detect, prevent and mitigate all possible problems that may occur in the microgrid as fast as possible so big-impact effects like blackouts, or the no supply of critical loads do not happen. Some of the action that this system allow us to take are: starting batteries, stopping loads, regulating power generation from the photovoltaic system by programming some protocols for the inverter, etc.

Therefore, it is the necessary to know the actual value of certain parameters of the generation and consumption systems so that the developed WAMPAC system can control them. In out particular case, the most important parameter is the power parameter based on the future studies set for the TIGON project. For this reason we focus on the following terms; power consumed by loads, power produced from renewable energy generation and power consumed or delivered by the storage systems.

In order to obtain the parameters to be monitored and managed, in most cases, the management systems communicate directly to each of the equipment’s converters by using a ModBus communication protocol. If direct communication with a converter is not possible, it is necessary to install a smart metering system (grid analyser). This would allow us to measure the required parameters and for the converter to communicate with the management system by using a communication protocol.

The monitoring and management system of CE.D.E.R.’s microgrid is based on three elements, each of them is dedicated to a specific task as detailed below, which can be summarised as communication, data recording and display of data to the user.

**Figure 12.**
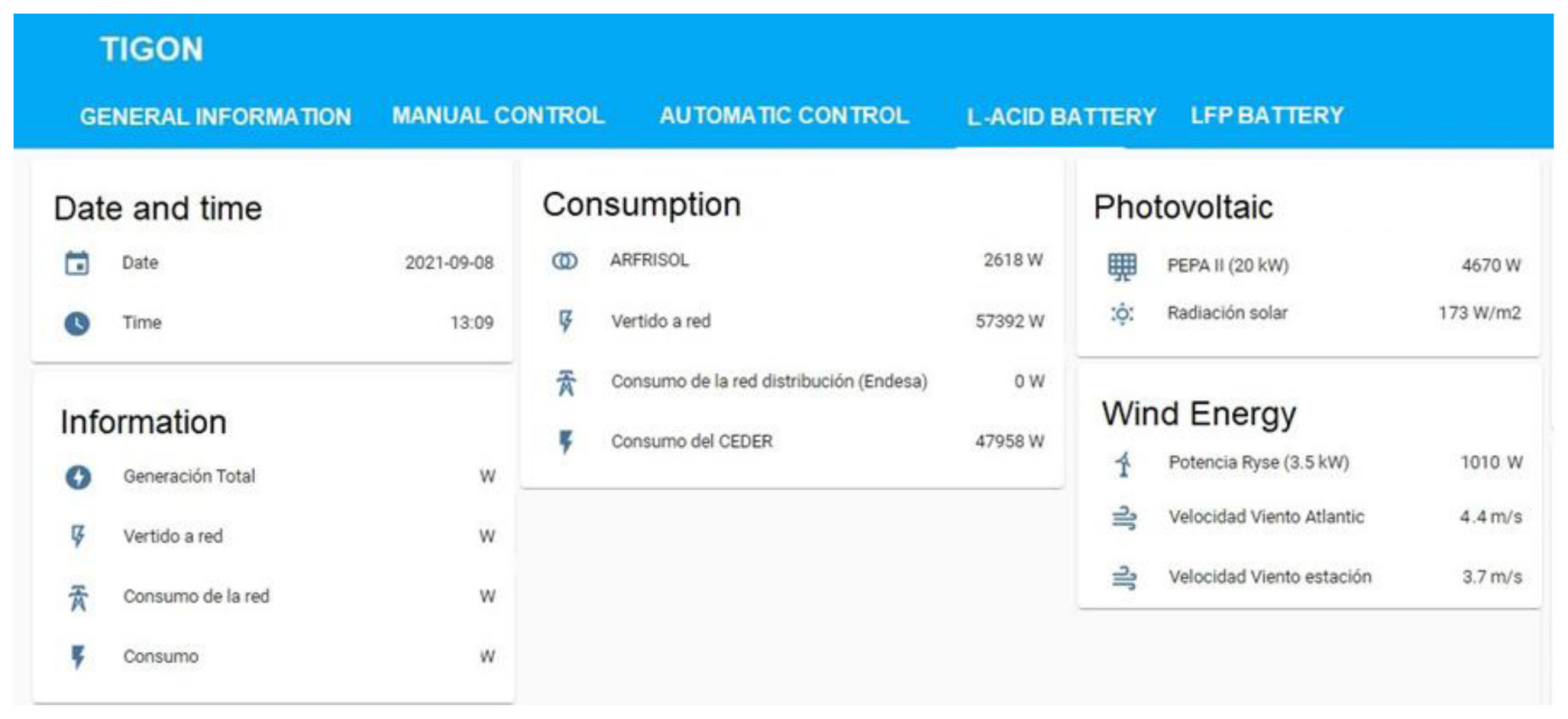
Example from energy management system (EMS).

- Communications block: based on NodRed. It integrates the different communication protocols of the generation, storage and consumption systems at CE.D.E.R. (Modbus, MQTT, HTTP, etc.).
- Database: it is based on a database management system called MariaDB. The latter is a relational database that allows us to storage data per second in real-time thanks to programmed events and calculations (minute average or 15-minute average, as the ones used by the energy distribution company that supplies CE.D.E.R.) through the corresponding queries.
This is important because, in addition to monitoring in real-time to make immediate decisions, it is of great interest to storage and analyse the data in order to stablish management strategies in the medium and long-term.
- Energy management system (EMS) with a human-machine interface based on the Home Assistant software (see Figure 12). This software allows monitoring all the necessary data from the equipment connected to the microgrid in real-time and giving instructions to each one. Furthermore, it is accessible from every point of the CE.D.E.R. communication network (meaning it is decentralized) and it can be accessed remotely with a smartphone application.

**Figure 13.**
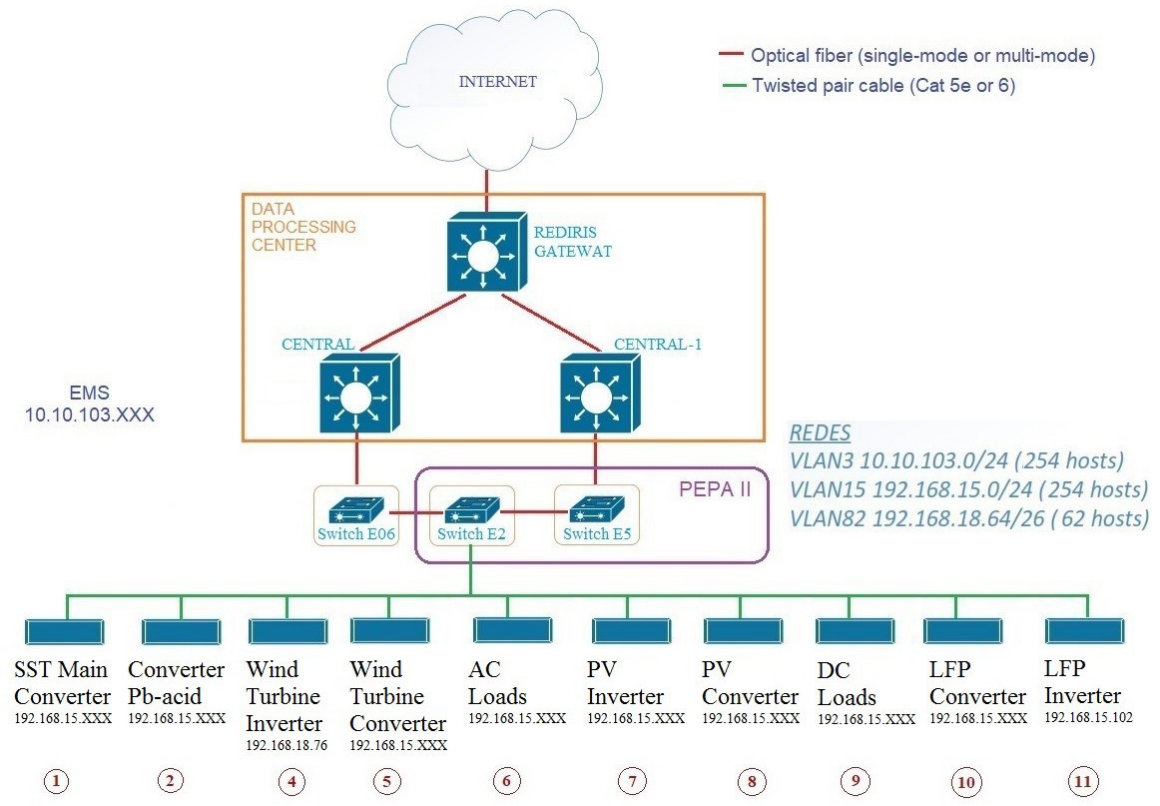
Microgrid communications network.

To achieve this, it is essential to connect all the elements of the microgrid as well as the management system to CE.D.E.R.’s data network. In this case, the equipment is connected to an ethernet data network (see Figure 13) that allows visualisation from any location.

Cybersecurity is closely linked to the microgrid’s management and control software. Due to the number of devices, entry points and protocols used, there is a bigger area susceptible to cyberattacks.

The modern cybersecurity market does not have the capacity to manage accurately such display for the entire power grid yet. Because of the latter, a member of the project is developing a security framework to provide a set of defensive measures in network attacking scenarios.

All of the above means that the operating mode and the cybersecurity system preparation is based on vulnerability evaluation and attack scenarios. The aim is to design a smart network architecture against malware. At the same time, the security performance will be analysed to reduce the impact of human errors and spear phishing campaigns.

## Results and discussion

The results obtained are those recorded with the operation of the generation and storage systems in AC on a daily basis in fortnightly periods. They are used to analyse their correct operation in AC until all the equipment necessary for DC operation is installed and they can start operating in this current mode, thereby obtaining data and being able to carry out comparative analyses and studies between the two types of current.

The following shows the data recorded during one day, specifically on 23 October 2022, of the power generated by the photovoltaic system, the wind system, the lead acid batteries and the LFP batteries, both in terms of charge and discharge.

As explained in the previous section, the parameters to be studied are recorded and saved in a database creating a file as shown in Table 1.

**Table 1.** Data recorded during 23 October 2022.

	PV system		Wind turbine		Battery systems			
Hour	Radiation [W/m ^2^]	PV System [W]	Wind [m/s]	RYSE E5 [W]	Lead-acid CHARGE [W]	Lead-acid DISCHARGE [W]	LFP CHARGE [W]	LFP DISCHARGE [W]
7:00	0	0	3,7	0	880	0	0	-19987
7:15	0	0	3,8	0	880	0	0	-19987
7:30	0	0	3,4	0	880	0	0	-19986
7:45	0	0	3,7	0	880	0	0	-19257
8:00	0	0	3,9	0	880	0	0	-356
8:15	0	1	4	0	880	0	0	0
8:30	1	31	3,8	0	880	0	0	0
8:45	7	147	3,7	0	880	0	0	0
9:00	21	385	2,7	0	880	0	0	0
9:15	22	430	3	0	880	0	0	0
9:30	24	472	4	0	880	0	0	0
9:45	30	612	4,3	0	880	0	0	0
10:00	50	955	3,4	0	664	0	0	0
10:15	64	1214	3,4	0	0	0	0	0
10:30	63	1300	3,9	0	0	0	0	0
10:45	89	1707	3,8	0	0	0	5510	0
11:00	120	2223	4,3	0	0	0	20136	0
11:15	148	2652	3,5	0	0	0	20137	0
11:30	160	3044	4,2	0	0	0	20139	0
11:45	172	3319	4,3	0	0	0	20142	0
12:00	237	5546	3,9	0	0	0	6039	0

The upper table shows a brief period of the day indicated for the different terms recorded, such as solar radiation, solar energy production, wind, wind energy production and the two battery systems, both charging and discharging, differentiated not only by their location in different columns, but also by the sign that precedes the number, positive if the system is charging and negative if it is in the process of discharging.

Based on these values, graphs are generated to analyse its operation on a daily basis and to conclude whether its operation is adequate or whether there is any deviation. If there is any deviation, a corrective action will be taken by the user to solve the problem.

**Figure 14.**
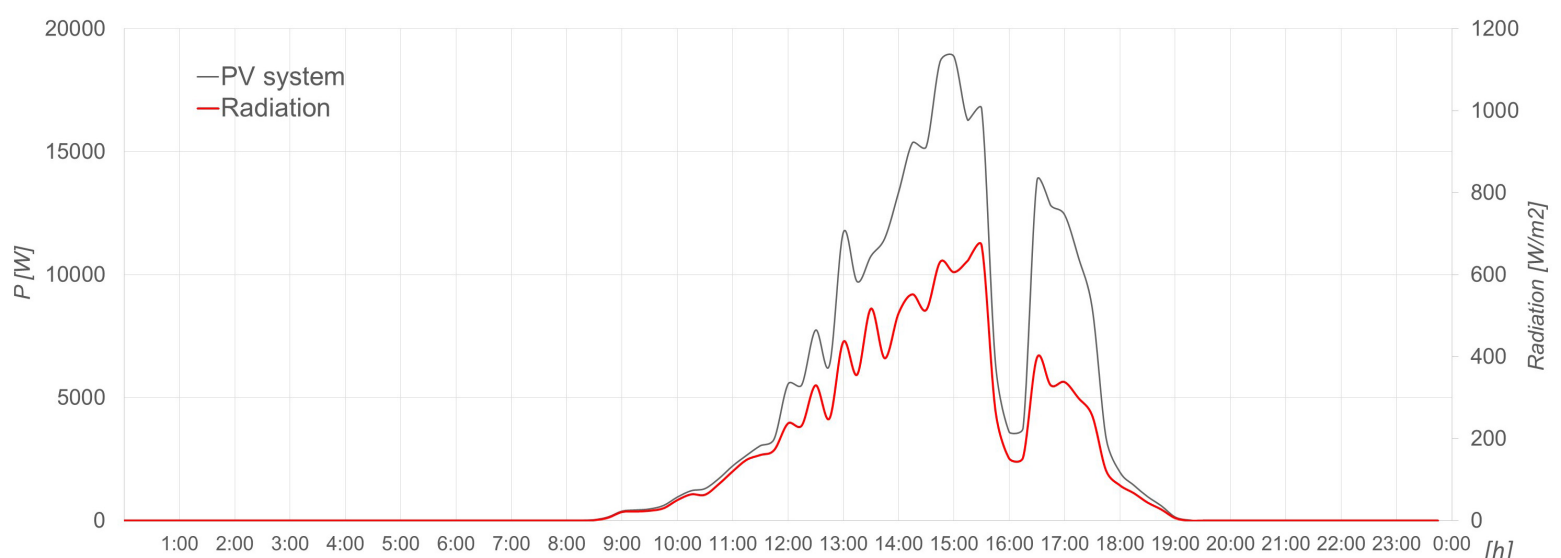
Solar radiation and solar energy production.

The following graphs (see Figure 14 – Figure 18) have been generated for 23 October 2022.

**Figure 15.**
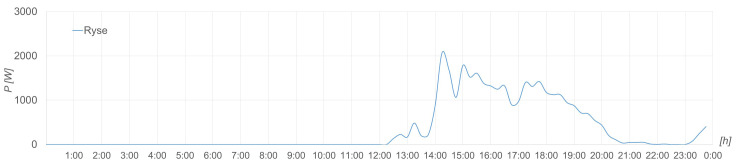
Wind energy production.

**Figure 16.**
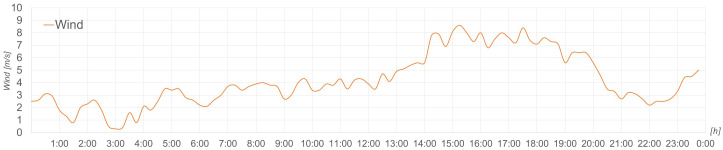
Wind recorded.

**Figure 17.**
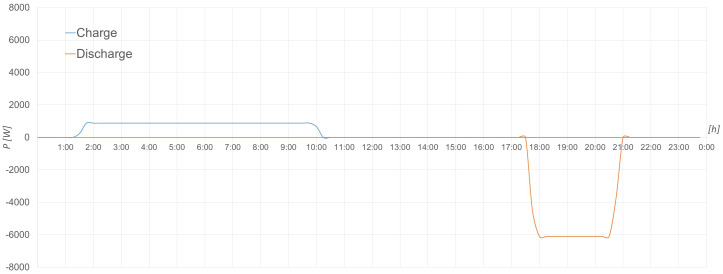
Charging and discharging process of lead-acid batteries.

**Figure 18.**
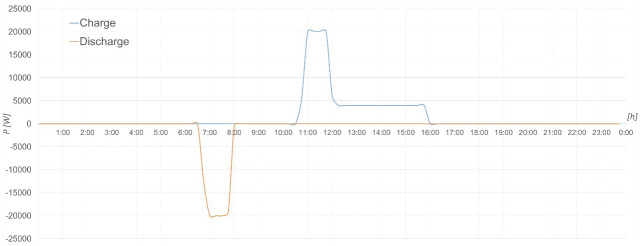
Charging and discharging process of LFP batteries.

In this first graph (see Figure 14) the solar radiation and the production of the photovoltaic system are represented. It can be seen that its operation is adequate as it follows the logical curve of an autumn day, both the radiation and the PV System that accompanies it.

The wind energy production is shown in Figure 15 in terms of power, which is correct taking into account the recorded wind speed in m/s shown in Figure 16 and the power curve of the wind turbine.

Focusing on the storage systems. Figure 17 shows the charging (blue line) and discharging (orange line) process of the lead-acid batteries based on the power indicated by the user through the EMS described above.

The same can be seen in Figure 18 with the LFP battery storage system, where as a detail different from the previous case is that here it has been charged with two different powers.

Both processes in the two storage systems have been carried out correctly, therefore their operation is as expected and appropriate for such equipment.

After making the individual analysis of each of the systems and checking that they are working properly, the only thing left to do is to wait for the installation of the rest of the elements in order to start the DC operation and its corresponding registers, as is currently being done.

## Conclusions and future work

The CE.D.E.R.-CIEMAT centre is a demonstration centre for the TIGON project and houses a microgrid with hybrid AC/DC architecture within its facilities.

Currently, in the second active year of the project, all generation, storage, and consumption systems are installed and connected as a microgrid as we know them today, in AC.

Recording the current data, the operation of the electrical microgrid and the instantaneous action taken on the equipment will allow us to carry out studies, comparisons, analysis of the variation in behaviour, costs, benefits and energy losses at equipment and global level. All of the latter will be compared to the hybrid microgrid registers.

All these studies and analyses are part of future work. To get there, we are researching and developing, as discussed in this document, SST, power converters, management systems, and cybersecurity.

Once all the components are installed and systems are created, we will then be able to start setting up the hybrid microgrid. With its operation and recording of values of energy generated at equipment and global level, energy consumed at global level, energy recorded before and after the converters, and equipment performance; we will be able to complete the proposed studies.

The conclusions of the project are intended to support the decision-making of grid operators and to steer actions towards decentralised hybrid microgrids. At present, the lack of DC microgrids prevents them from being a promising solution for future distribution grids and becoming a commonly used technology.

In this way, a positive impact is achieved with the demonstration of the project in our facilities. This project intends to set the bass of DC-based microgrids and to envision them as an optimal solution and future horizon to be addressed. The tools to achieve these goals are the ease of replicability with the technology developed in this project, as well as the management and cybersecurity systems implemented for both operators and consumers. In addition, some other goals are improving generation efficiency and facilitating the energy transition, following the decarbonisation targets set by the European Union.

## Data Availability

No data are associated with this article.
