# Supplementary material for: Hybrid AC/DC architecture in the CE.D.E.R.-CIEMAT microgrid: demonstration of the TIGON project
Source: Open Res Eur. 2024 Jan 9;2:123. Originally published 2022 Oct 26. [Version 2] doi: 10.12688/openreseurope.15154.2 (PMC10804056; doi:10.12688/openreseurope.15154.2)
Supplement: Peer review history

# Reviewer report: Reviewer response for version 2

**DOI:** 10.21956/openreseurope.18459.r37348

**Published:** 2024-01-22

**Authors:** Ehab H.E. Bayoumi

**Affiliations:** 1 The British University in Egypt, El Shorouk City, Cairo Governorate, Egypt

The authors have made all the requested comments. No further comments from my side. (Accepted)

Is the case presented with sufficient detail to be useful for teaching or other practitioners?

Partly

Is the work clearly and accurately presented and does it cite the current literature?

Yes

If applicable, is the statistical analysis and its interpretation appropriate?

No

Are all the source data underlying the results available to ensure full reproducibility?

No

Are the conclusions drawn adequately supported by the results?

Partly

Is the background of the case’s history and progression described in sufficient detail?

Yes

Reviewer Expertise:

High-performance ac machines, power quality, switching power converters in smart and microgrids, and nonlinear control applications in power electronics, smart grids, and electric drive systems.

I confirm that I have read this submission and believe that I have an appropriate level of expertise to confirm that it is of an acceptable scientific standard.

# Reviewer report: Reviewer response for version 1

**DOI:** 10.21956/openreseurope.16392.r33386

**Published:** 2023-08-17

**Authors:** Othmane ABDELKHALEK, Mohamed Amine Hartani

**Affiliations:** 1 Tahri Mohamed University Béchar, Béchar, Béchar, Algeria; 2 University Ahmed Draia, Adrar, Algeria

The paper provides a clear and concise overview of the TIGON project, its objectives, and the proposed microgrid design. It effectively communicates the significance of the project and the potential benefits of implementing a DC-based hybrid grid architecture.

 Overall, this paper seems to be a short presentation of a power system belonging to a recently realized project (02 years).

 Besides, it is fuzzy to understand this paper without significant and clear contributions/novelty/clear results with discussions and meaningful conclusions in light of the literature review. The results and discussion section are missing; thus, the power system is a black box and should be identified.

 However, many issues have been observed to be solved aiming to improve the paper content and presentation as well.

 1) The abstract is well-written, apart from that it does not contain the main findings and conclusions. This confirms how important your proposed project is, especially using experimental validation.

 2) The keywords should be arranged alphabetically as follows:  

*Direct current (DC), DC/AC converters, DC/DC converters, Hybrid microgrid, Low voltage Direct current (LVDC), and Medium Voltage Direct Current (MVDC).*

 3) The authors stated in the 3rd paragraph of the introduction that: ‘ ***This conversion produces energy losses, both in the converter, producing a total energy loss of approximately 10–25%*** *^1,3,4^ **, and in the transport from the point of generation to the point of consumption***.’

 This information was included in reference **01**, but unfortunately not in reference **03**. Besides, in reference **04**, it was stated that: ‘ ***The goal was to use this clean energy source directly whenever it was available and not invert it to ac power, avoiding the typical 7–15% energy loss from the conversion process.*** ’

**The question is:** which statistic is acceptable or real? The authors should pay attention to their citations when providing sensitive statistics to readers, please solve this issue with reliable references or discuss the different opinions of cited authors.

 4) The whole introduction cites only 6 references, resulting in weak content of the introduction. The authors should present their proposed contributions in light of the literature and what was already achieved by other authors within the **last 5 years**.

 5) Which standard do authors use to specify that **240V _DC_** belongs to Medium DC voltage (MVDC)? Please, check these standards and verify:

- https://webstore.iec.ch/preview/info_iec61140%7Bed4.0.RLV%7Den.pdf
- The International Electrotechnical Commission (IEC) Standard IEC 61140:2016 defines LV as 0 to 1000 V _AC_ RMS or 0 to 1500 V _DC_ . Other standards such as IEC 60038 define *supply system LV* as voltage in the range of 50 to 1000 V _AC_ or 120 to 1500 V _DC_ in *IEC Standard Voltages*, which defines power distribution system voltages around the world.

6) Figure 1 displays the Diagram of AC/DC hybrid microgrid architecture, however, it contains several manual switches to manage the circulation of resources currents or powers flowing between the generation and consumption sides. In fact, the real and effective management requires controllable switches rather than manual ones. I recommend to use these switches in the future work.

 7) The paper didn’t include any details about the energy management used method (organigram).

Is the case presented with sufficient detail to be useful for teaching or other practitioners?

Partly

Is the work clearly and accurately presented and does it cite the current literature?

Partly

If applicable, is the statistical analysis and its interpretation appropriate?

I cannot comment. A qualified statistician is required.

Are all the source data underlying the results available to ensure full reproducibility?

Partly

Are the conclusions drawn adequately supported by the results?

Yes

Is the background of the case’s history and progression described in sufficient detail?

Partly

Reviewer Expertise:

DC microgrids; LVDC microgrid; Smart Grids; Renewable Energies; Power quality; Electrical power system

We confirm that we have read this submission and believe that we have an appropriate level of expertise to confirm that it is of an acceptable scientific standard, however we have significant reservations, as outlined above.

# Response

**Published:** 2023-12-29

**Authors:** Paula Peña-Carro

**Affiliations:** CIEMAT, Spain

Thank you for your review. 

 We have tried to respond to all your suggestions. I would like to explain about your 5th comment because I think you misunderstood what we wanted to explain in the document and in order to understand it correctly I will try to explain it below.

 In the case of low/medium and high voltage standards according to AC and DC voltage. It is not being indicated that 240 Vdc are considered MVDC, what we are trying to transmit is that the batteries are located on the medium voltage line, that is why reference is made to MVDC and that by means of a converter this MVDC voltage is adapted to that of the batteries, which work in LVDC. This management is motivated to create a direct working line from MVDC to LVDC.

# Reviewer report: Reviewer response for version 1

**DOI:** 10.21956/openreseurope.16392.r33384

**Published:** 2023-07-21

**Authors:** Daniele Bosich

**Affiliations:** 1 University of Trieste, Trieste, Piazzale, Italy

The scientific paper offers a starting discussion on a very interesting topic like hybrid AC/DC architecture. More details on the intial desription are necessary (why hybrid AC/DC architecture? Advantages? Weak points? Solutions?). In my opinion the work is good, but more attention on results is also necessary. The general description is ok, but I suggest to add a small detailed description on the power converters. Also the control section needs additional remarks to encourage the reader comprehension. A final section on transients in presence of perturbation can increase the paper's importance and value. Also please largely review the references. Now they are insufficient. I see something like 20-25 references for this kind of paper.

Is the case presented with sufficient detail to be useful for teaching or other practitioners?

Partly

Is the work clearly and accurately presented and does it cite the current literature?

No

If applicable, is the statistical analysis and its interpretation appropriate?

Partly

Are all the source data underlying the results available to ensure full reproducibility?

Partly

Are the conclusions drawn adequately supported by the results?

Partly

Is the background of the case’s history and progression described in sufficient detail?

Yes

Reviewer Expertise:

Microgrid, DC control, power management system, stability

I confirm that I have read this submission and believe that I have an appropriate level of expertise to confirm that it is of an acceptable scientific standard, however I have significant reservations, as outlined above.

# Response

**Published:** 2023-12-29

**Authors:** Paula Peña-Carro

**Affiliations:** CIEMAT, Spain

First of all, thank you for your review.

 We have tried to respond to all your comments with the exception of the power converters, this is because we do not have more information than detailed in the document. We are not aware of more specific design terms than those discussed. We are also unable to provide details on the transition related to this type of equipment as we do not have data on this aspect.

# Reviewer report: Reviewer response for version 1

**DOI:** 10.21956/openreseurope.16392.r33382

**Published:** 2023-07-18

**Authors:** Ehab H.E. Bayoumi

**Affiliations:** 1 The British University in Egypt, El Shorouk City, Cairo Governorate, Egypt

This article describes the project's demonstrative progress toward its goal of optimizing the network's performance via a hybrid grid based on intelligent DC power sources, as well as the predetermined goals that can be accomplished by incorporating a DC-based microgrid with smart grid architecture into the existing power infrastructure. In this report, some comments should be done, such as:

1. No result is taken from the implemented parts in the system to show how they are practically implemented successfully.
2. Some statements have to be corrected as:
  - Plain language summary section, we can not generalize that wind energy produced DC depends on the generator connected to the wind system.
  - Plain language summary section, most of the consumption is made in DC (incorrect), Most domestic consumption is AC.
  - Page 4, Col 1, paragraph. 2, vary the power of all the equipment instantaneously. Each equipment device has its own rating and Power which has to be supplied, there is no varying in power to equipment,
  - Many references are cited incorrectly as in the Introduction section, page 3, col 2, paragraphs 1 and 2.
  - Figure 1, circuit diagram for the hybrid microgrid architecture, how the 230 V ac is connected to 400 V ac directly (blue line on the left side of the diagram)? 
3. No statistical tables or diagrams to show the needs of implementing this project, just text.

Is the case presented with sufficient detail to be useful for teaching or other practitioners?

Partly

Is the work clearly and accurately presented and does it cite the current literature?

Yes

If applicable, is the statistical analysis and its interpretation appropriate?

No

Are all the source data underlying the results available to ensure full reproducibility?

No

Are the conclusions drawn adequately supported by the results?

Partly

Is the background of the case’s history and progression described in sufficient detail?

Yes

Reviewer Expertise:

High-performance ac machines, power quality, switching power converters in smart and microgrids, and nonlinear control applications in power electronics, smart grids, and electric drive systems.

I confirm that I have read this submission and believe that I have an appropriate level of expertise to state that I do not consider it to be of an acceptable scientific standard, for reasons outlined above.

# Response

**Published:** 2023-12-29

**Authors:** Paula Peña-Carro

**Affiliations:** CIEMAT, Spain

Thank you for your review in the first place. 

 We have tried to respond to all your suggestions so that the article is more complete and hope that the review is to your pleasure.
